# Report on Proceedings of the Ninth Annual European CME Forum, Amsterdam, the Netherlands, November 2016

**DOI:** 10.1080/21614083.2017.1302672

**Published:** 2017-04-03

**Authors:** Ron Murray

**Affiliations:** ^a^ Independent CME/CPD Consultant, Pickering, UK

**Keywords:** CME/CPD, quality, evaluation, engagement, outcomes, interprofessional, innovation

## Abstract

Participants from as far afield as Australia and North America convened in Amsterdam, the Netherlands between 9 and 11 November 2016 to attend the Ninth European CME Forum (ECF). The meeting combined panel discussions with a range of presentations on topics such as high quality learning, current challenges in CME and future trends. However, the bulk of the forum was taken up with interactive workshops under three different track headings: Development, Educational Design and the CME Environment. Attendees also heard from a group of CME pioneers in a pre-conference session on the history of CME on both sides of the Atlantic Ocean.

A truly global array of participants from as far afield as Australia and North America met in Amsterdam, the Netherlands on 9–11 November 2016 for one of the largest gatherings of the ECF, now in its ninth year. The meeting format combined thought-provoking presentations and panel discussions with interactive forum workshops tracked under the headings of Development, Educational Design and the CME Environment.

Day 1 began with a pre-conference session that provided a perspective on the history of CME regulation and accreditation from four of CME’s “founding fathers” from Europe and USA. Drs Cees Liebbrandt and Len Harvey representing The European Union of Medical Specialists (UEMS) outlined the formation of UEMS and the European Accreditation Council for CME (EACCME) following the Charter on European CME in 1994 and the subsequent negotiations with the American Medical Association (AMA) for mutual recognition of CME credits between Europe and the USA.

Dr Dennis Wentz described his role in negotiating the reciprocal agreement on behalf of the AMA and pointed out the existence of global standards for Continuing Professional Development (CPD) set out by the World Federation for Medical Education (WFME) and updated in 2015 (http://wfme.org/standards/cpd).

Dr Wentz went on to present a paper on behalf of Lewis Miller, founder of both the Alliance for Continuing Education in the Health Professions (ACEhp) and the Global Alliance for Medical Education (GAME) who was unable to attend in person due to ill-health. He traced the history of the formation of the Accreditation Council for Continuing Medical Education (ACCME) in 1981 to the current approach in North American CME where interprofessional activities focus much more on measuring outcomes in terms of changes in healthcare professionals’ behaviour and patient care improvement.

The main conference was introduced by Eugene Pozniak, ECF’s co-founder and Programme Director, who highlighted the milestones in European CME during the previous 12 months, including the accreditation status granted by ACCME to the first non-US organisation, namely SIYEMI Learning. This status provided the means for this year’s forum to be designated for *AMA PRA Category 1 Credits™*.

During the opening session, Jonas Nordquist of the Karolinska Institutet illustrated results from research on learning at undergraduate and postgraduate level in medicine that could be translated into strategies for success in CME. He noted the importance of the design of learning spaces to bolster infrastructure for high quality learning and provided examples of networked learning landscapes.

The final session of Day 1 was a panel discussion chaired by health journalist Jacqui Thornton who elicited a wide range of discussion points on European CME issues from an international panel representing Germany, UK, USA and Switzerland. The topics covered included:

- smaller scale “quality” CME activities in Europe involving 25–50 people;
- industry having a role in content development;
- collaboration between providers to produce CME activities;
- funding of international programmes;
- the problems associated with obtaining data for outcomes measurements
- funding sources e.g. self-pay, employers;
- lack of overarching standards; and
- higher level outcomes measurements.

Day 2 focused on a series of concurrent facilitated workshops that gave delegates the opportunity to interact in smaller groups. Three different tracks were available for participants:

- Development;
- Educational Design; and
- CME Environment.

## Development

The three development workshops were presented by delegates from Europe and USA.

- Diana van Brakel of Kenes Education and Celeste Kolanko of PCM Scientific provided case-based scenarios to illustrate four pillars of quality and effective CME as defined by the good CME practice group (http://goodcmepractice.eu/) These are Effectiveness, Appropriate Education, Transparency and Balance.
- Thomas Kellner of UCB Pharmaceuticals coordinated a workshop with colleague Audrey Noble and her husband Marc Blore. They outlined a process to develop learning outcomes based on a gap analysis that emphasised the patient’s perspective, as illustrated by Marc’s status as a person living with Parkinson’s disease. The next steps involved classifying gaps into learning domains, linking learning outcomes with professional competencies and making an appropriate choice of educational format.
- Julie Simper of International CME-CPD Consulting and Sophie Wilson of International Medical Press and both members of the Good CME Practice group also used the group’s principles of quality and effectiveness to show participants how to “Navigate Compliance Waters”. Using case studies they illustrated methods of being compliant within the framework of regulatory and accreditation criteria in accordance with the individual provider’s specific circumstances.

## Educational design

Only two workshops were presented in this track due to the illness of one workshop leader.

- Kate Regnier, Executive Vice-President of ACCME conducted a workshop on evaluation of CME by posing the fundamental question, “what are you trying to change?” in terms of knowledge, competence (skill/strategy/attitude), at individual or team level or at community level such as patient outcomes or population health.

The main thrust of this workshop was to have the participants consider how to evolve educational activities from knowledge transfer to creative and social engagement that promotes cognitive/skill development and better patient outcomes.

b.Kathy Chappell of ANCC was joined by Dr Helena Filipe of the International Council of Ophthalmology, Lawrence Sherman of TOPEC Global and Dr Mark Westwood of St. Bartholomew’s Hospital in London to bring a transatlantic perspective via role play and group discussion to consider how to design an effective interprofessional CME activity. The main strategies suggested were:

- start with an icebreaker that has nothing to do with planning Continuing Education;
- use first names, not professional titles;
- mix professions by deliberate seating;
- maintain a patient or problem-centric focus;
- be prepared to handle professional hierarchy behaviours (dominating conversation, passivity); and
- mix up tables/conversation.

## CME environment

The third workshop track comprised the following:

- Representatives of UEMS-EACCME led by Secretary General Professor Vassilios Papalois provided workshop participants with an outline of the quality criteria set forth by EACCME, the application process and the requirements for activity standards i.e. that CME activities should:
- be international;
- contain defined objectives;
- describe learning methods;
- be scientifically valid and balanced; and
- fulfil the educational objectives.

The proposed EACCME-2 criteria were also outlined that include:

- a faster and more efficient application and review process;
- an updated IT platform to support the process;
- consideration of new educational formats;
- recognition of authorship and teaching as eligible CME/CPD activities;
- “trusted provider” status;
- quality control of events; and
- an expansion of interprofessional learning.
  b.Industry representatives from Belgium, Germany, Austria and USA stimulated much discussion among participants by presenting for consideration a potential role for the pharmaceutical industry in providing high quality complementary education to help healthcare professionals improve practice to benefit patients.

It was suggested that quality assurance could be maintained by appropriate monitoring, governance and evaluation to eliminate bias.

c.Lisa Sullivan from Australia, representing GAME, was joined by Vaibhav Srivastava from India and Dr Alvaro Margolis via Skype from Uruguay to describe the characteristics of CME activities in Asia, India and Latin America. They highlighted similarities and differences between these regions and North America and Europe. The main take-home messages related to the variations in regulatory practices, the increasing emphasis on internet-based education, particularly in Latin America, and the lack of needs-based educational activities in much of Asia. The presenters also noted the important influence of different cultures that affect the way CME activities are planned and implemented in these regions and discussed the distinct possibilities for transferability of CME activities across language and cultural barriers.

The morning and afternoon workshop sessions were interspersed with a panel discussion led once again by Jacqui Thornton. The panellists from Canada, UK, Portugal, Georgia and Austria relayed many of the key points from each of the morning workshops to the assembled participants.

Day 3 was introduced with details of the move of the *Journal of European CME* to the publishing house of Taylor and Francis and the opportunities for authorship via this diamond open-access journal (www.jecme.eu).

The first morning session dealt with novel educational approaches from the perspective of presenters from Canada, USA and Belgium.

The opening presentation provided an insight into the role of the patient in CME/CPD, illustrated by a patient’s own description of how an organisation such as Lupus Europe promotes patient involvement.

Next, a new code of ethical standards for the medical technology industry in Europe was described that brings traditional interactions between industry and healthcare professionals under more detailed scrutiny. This has led to some misgivings from large medical societies used to receiving funding via industry sponsorship. Nevertheless, the code is currently being rolled out and may well affect the way that some traditional CME activities are planned and implemented in Europe.

A reassuring note was provided by Cyndi Grimes of WebMD Global who stated that a significant majority of their global learners (around 85%) using linked learning assessments (LLAs) were primarily seekers of knowledge in short timeframes (less than 30 min) rather than credit seekers. Moreover, activities that are case-based, mixed media, and interactive have proven to be the most effective and popular format with participants.

The final presentation from Jennifer Gordon of the Royal College of Physicians and Surgeons of Canada described some of their efforts to encourage interaction in CME activities. These included the creation of communities of practice, simulation and gamification.

A wrap-up session chaired by Jacqui Thornton considered “What the Future Holds” and participants heard from Dr Graham McMahon, CEO of ACCME, Professor Vassilios Papalois of UEMS, and Marie-Claire Pickaert of EFPIA. The discussion led to some vigorous dialogue with the audience and some memorable snippets were:

“We are seeing a move away from valuing credit rather than learnings”

“We need to see beyond the bureaucracy of accreditation”

“Collaboration is paramount and industry should have a role”

“The science from industry should be incorporated into CME but not in a promotional way”

“CME needs to be elevated as a profession”

“Healthcare professionals need a safe place for learning and it can be local”

“We want to be active players and not accreditation police”

Lawrence Sherman’s “unsession” once again rounded off the 9th ECF raising points for consideration such as:

- funding sources;
- the paucity of collaboration efforts in European CME; and
- the role of ECF – should it be a membership body?

Full details of the presentations and support materials may be accessed at the European CME Forum website: http://europeancmeforum.eu

The Twitter stream for the meeting can also be obtained at https://twitter.com/hashtag/9ecf

The 10th European CME Forum will take place in Dublin, Ireland between 8 and 10 November 2017.

